# Aspalatone Prevents VEGF-Induced Lipid Peroxidation, Migration, Tube Formation, and Dysfunction of Human Aortic Endothelial Cells

**DOI:** 10.1155/2017/2769347

**Published:** 2017-01-24

**Authors:** Himangshu Sonowal, Pabitra B. Pal, Kirtikar Shukla, Kota V. Ramana

**Affiliations:** Department of Biochemistry and Molecular Biology, University of Texas Medical Branch, Galveston, TX 77555, USA

## Abstract

Although aspalatone (acetylsalicylic acid maltol ester) is recognized as an antithrombotic agent with antioxidative and antiplatelet potential; its efficacy in preventing endothelial dysfunction is not known. In this study, we examined the antiangiogenic, antioxidative, and anti-inflammatory effect of aspalatone in human aortic endothelial cells (HAECs). Specifically, the effect of aspalatone on VEGF-induced HAECs growth, migration, tube formation, and levels of lipid peroxidation-derived malondialdehyde (MDA) was examined. Our results indicate that the treatment of HAECs with aspalatone decreased VEGF-induced cell migration, tube formation, and levels of MDA. Aspalatone also inhibited VEGF-induced decrease in the expression of eNOS and increase in the expression of iNOS, ICAM-1, and VCAM-1. Aspalatone also prevented the VEGF-induced adhesion of monocytes to endothelial cells. Furthermore, aspalatone also prevented VEGF-induced release of inflammatory markers such as Angiopoietin-2, Leptin, EGF, G-CSF, HB-EGF, and HGF in HAECs. Thus, our results suggest that aspalatone could be used to prevent endothelial dysfunction, an important process in the pathophysiology of cardiovascular diseases.

## 1. Introduction

Endothelial cells that form new blood vessels play an important role in the pathophysiology of cardiovascular diseases (CVD) [1]. Although, normal endothelial cell function is involved in various physiological processes such as angiogenesis, embryonic development, wound healing, and tissue remodeling, abnormal endothelial function could lead to increased vascular complications, cancer growth, and metastasis [2–4]. Factors that interrupt normal endothelial cell functions such as injury, lipid infiltrations, oxidative stress, and inflammatory response could lead to endothelial dysfunction [5, 6]. Several studies have also shown that the widespread vascular endothelial activation, dysfunction, and damage lead to multiple organ failure during severe bacterial infections and in sepsis [7]. Furthermore, endothelial function that leads to angiogenesis is involved in the tumor development, survival, invasion, and metastasis [3, 8]. Several studies have shown the significance of vascular endothelium in various disease pathologies and identified several agents that can prevent endothelial dysfunction as potential agents to control CVD [2, 4, 6, 9].

During pathological conditions, increased oxidative stress generates reactive oxygen species (ROS) via NAPDH oxidase/mitochondrial pathways. Increased ROS formation leads to peroxidation of lipids and release toxic lipid aldehydes such as malondialdehyde (MDA) and 4-hydroxynonenal. The lipid aldehydes could act as secondary signaling intermediates to alter signaling pathways responsible for cellular equilibrium leading to change in the endothelial cell phenotype, increase proinflammatory gene expression, tissue damage, and endothelial dysfunction [10–13]. Increased expression of FGF and VEGF during oxidative stress induces endothelial dysfunction [14–17]. Besides CVD, VEGF has also been shown to be involved in endothelial dysfunction in patients with preeclampsia and chronic kidney disease [18, 19]. Several VEGF inhibitors have been synthesized and tested as potential CVD drugs. However, some of the VEGF inhibitors have been shown to cause side effects such as increased endothelial toxicity, predisposing to thrombosis and hypertension [20–23]. Besides several synthetic anti-VEGF agents, several reports also suggest the significant use of various antioxidants in the prevention of endothelial dysfunction [24, 25]. Antioxidants such as curcumin, quercetin, resveratrol, and N-acetylcysteine have been shown to prevent endothelial dysfunction in preclinical studies [24, 26–29]. Although, some of these antioxidants have gone to clinical trials for the treatment of CVD, they are not as effective as they are in experimental animals [30, 31]. Therefore, alternative antioxidant regimens are required to control endothelial dysfunction.

Aspirin (acetylsalicylic acid) is a frontline antipyretic and anti-inflammatory agent with potent antithrombotic activity, saving millions of lives in patients with CVD [32, 33]. Besides CVD, aspirin has also been shown to decrease the rate of cancer growth and metastasis and improved overall survival rate in patients [34–36]. Furthermore, aspirin has been shown to be effective in inhibiting the release of various inflammatory cytokines responsible for CVD [37, 38]. However, side effects such as a gastrointestinal insult have been reported with higher doses of aspirin use [39]. Even with short term administration, aspirin reportedly exhibited both gastric and duodenal mucosal damage [40]. Thus, to overcome these problems, different formulations of antithrombotic agents with similar therapeutic potential but lesser side effects have been developed.

Aspalatone is a potent antioxidant synthesized by the esterification of acetylsalicylic acid (aspirin) and maltol [41–43]. Compared to aspirin, aspalatone has been reported to show negligible gastrointestinal damage and possesses potent antithrombotic activity [41, 44, 45]. Aspalatone also inhibits collagen-induced platelet aggregation in vitro and in vivo (IC_50_ = 180 *μ*M) [45]. However, the effect of aspalatone in preventing endothelial dysfunction is not known. In the present study, we examined the effect of aspalatone on VEGF-induced endothelial cell growth, migration, tube formation, and monocyte adhesion. Our results indicate that aspalatone prevents VEGF-induced endothelial cell dysfunction by preventing VEGF-induced increase in the lipid peroxidation, inflammatory and adhesion maker expression, and iNOS and VCAM-1 expression and decrease in eNOS levels. Thus, our results suggest that aspalatone, a novel derivative of aspirin, could be used for the prevention of endothelial dysfunction and its associated complications.

## 2. Material and Methods

### 2.1. Materials

Aspalatone was purchased from Cayman Chemical Company. Endothelial Cell Medium (ECM) was obtained from ScienCell™ Research Laboratories. PBS, penicillin/streptomycin solution, and Trypsin/EDTA were obtained from Invitrogen. FBS was obtained from Gemini Bio-Products. MTT was obtained from Sigma. RIPA buffer was obtained from Santa Cruz Biotechnology. Vascular Endothelial Growth Factor (VEGF-165) and antibodies against VCAM-1, ICAM-1, eNOS, and GAPDH were obtained from Cell Signaling Technologies. Antibody against iNOS was obtained from Abcam. CM-H_2_DCFDA was obtained from Molecular Probes, Invitrogen. Malondialdehyde (MDA) detection kit was obtained from Oxis Research. Human Milliplex angiogenic cytokine multiplex kit was obtained from Millipore. All other reagents and chemicals used were of analytical grade and were obtained from Sigma.

### 2.2. Cell Culture

Primary human aortic endothelial cells (HAECs) were obtained from ScienCell™ Research Laboratories and were grown in complete Endothelial Cell Medium (ECM) supplemented with endothelial growth factors along with 5% FBS and 1% penicillin/streptomycin. All the cells were maintained at 37°C in a humidified atmosphere of 5% CO_2_.

### 2.3. Measurement of Cytotoxicity

HAECs (3000 cells/well) were growth-arrested with 0.1% serum containing different concentrations of aspalatone (25–200 *μ*M) without or with VEGF (10 ng/mL). After 24 h, cell viability was determined by MTT assay.

### 2.4. Cell Migration Assay

HAECs were seeded in 12-well plates and allowed to form a confluent monolayer. The cells were then growth-arrested overnight in 0.1% FBS containing media. A longitudinal uniform scratch at the center of the monolayer was made with a 10 *μ*L sterile pipette tip carefully, and the monolayer was washed 3x with serum-free media. HAEC media with 0.1% FBS and VEGF (10 ng/mL) containing vehicle or aspalatone (50 *μ*M) was added to the wells. The wells were photographed at 0 h and 18 h. Percentage changes in the cell migration were calculated by formula (Width_0 hr_−Width_18 hr_)/Width_0 hr_ × 100.

### 2.5. In Vitro Capillary Tube Formation Assay

HAEC tube formation assay was performed by using an in vitro angiogenesis kit from EMD Millipore following manufacturer's instructions. Briefly, 50 *μ*L of diluted ECM matrix solution was added to each well of 96-well plate and incubated at 37°C to allow the matrix to solidify. Growth arrested HAECs were harvested and then seeded carefully without disturbing the solidified matrix at 1 × 10^4^ cells/well in the absence and presence of aspalatone (10 *μ*M, 20 *μ*M, 50 *μ*M, and 100 *μ*M) and incubated for additional 18 h. Photographs were taken and the length of the capillary network was quantitated as described earlier [46, 47].

### 2.6. Monocyte-Adhesion Assay

In vitro monocyte cell adhesion assay was performed as described earlier [46]. Briefly, HAECs were seeded in 96-well plates at a density of 3000 cells/well and subconfluent cells were treated with aspalatone (50 *μ*M) overnight. After incubation, the cells were rinsed with serum-free media and THP-1 cells were added in a ratio of 1 : 3 (HAEC : THP-1) in the absence or presence of aspalatone and VEGF (10 ng/mL) for another 18 h. At the end of the incubation period wells were washed and 100 *μ*L of media containing MTT was added and incubated for another 3 h. Formazan crystals were dissolved by the addition of DMSO and absorbance was recorded at 570 nm using a plate reader.

### 2.7. Measurement of ROS Accumulation in HAECs

Intracellular ROS accumulation was measured by flow cytometry using CM-H_2_DCFDA. CM-H_2_DCFDA is a chloromethyl derivative of H_2_DCFDA used for measuring reactive oxygen species (ROS) in cells. To analyze ROS levels, the cells were treated for 18 h with VEGF (10 ng/mL) in the absence or presence of aspalatone (50 *μ*M). After the incubation period, the cells were stained with CM-H_2_DCFDA for 5 min, harvested, and analyzed immediately by a Flow Cytometer (BD LSRII Fortessa). Data analysis was performed using Flow Jo (Treestar, Ashland, OR, USA) and represented as fold change of Mean Fluorescence Intensity (MFI) compared to control.

### 2.8. Analysis of MDA Levels in HAECs

Lipid peroxidation marker, MDA, levels were measured in HAECs by using a kit from Oxis International Inc., following manufacturer's instructions. Briefly, the cells were treated for 18 h with VEGF (10 ng/mL) in the absence or presence of aspalatone (50 *μ*M). The cells were then washed with PBS 2x and lysed in PBS by sonication. Cell debris was removed by centrifugation at 3000 ×g for 10 minutes at 4°C. The supernatant was used in the assay as per the instructions and the absorbance was recorded at 586 nm using a plate reader. Total MDA levels (*μ*M) were calculated based on the standard curve and normalized to protein levels.

### 2.9. Analysis of Inflammatory Cytokines Secreted by HAECs

Analysis of various cytokines secreted by HAECs treated with VEGF (10 ng/mL) in absence or presence of aspalatone (50 *μ*M and 100 *μ*M) was analyzed by using a Human Angiogenesis/Growth Factor Magnetic bead panel kit from Millipore following manufacturer's instructions. Briefly, the culture media was collected, concentrated by lyophilization, and incubated with the labelled magnetic beads. After overnight incubation, the beads were counterstained with Streptavidin-Phycoerythrin and analyzed with a Luminex™ analyzer from Millipore. The results are expressed as pg/mL based on the standard curve generated with the standards.

### 2.10. Western Blot Analysis

Equal amounts of cell lysates (40 *μ*g) were loaded and separated on 12% SDS-polyacrylamide gels and transferred to polyvinylidene difluoride (PVDF) membranes. Membranes were then blocked with 5% nonfat dried milk and incubated with the specific primary antibodies at 4°C overnight followed by incubating with the specific secondary antibodies. Immunolabeling was detected using SuperSignal™ West Pico Chemiluminescent Substrate (ECL) from Thermo Scientific following manufacturer's instructions. The membranes were stripped with Restore™ PLUS stripping buffer from Thermo Scientific following manufacturer's instruction for reprobing with other antibodies or GAPDH.

### 2.11. Statistical Analysis

Data presented as mean ± SD and *p* values were determined by using an unpaired Student's* t*-test from Microsoft Office Excel 2007 software. *p* < 0.01 was considered as statistically significant.

## 3. Results

### 3.1. Effect of Aspalatone on HAECs Viability

**Figure 1:**
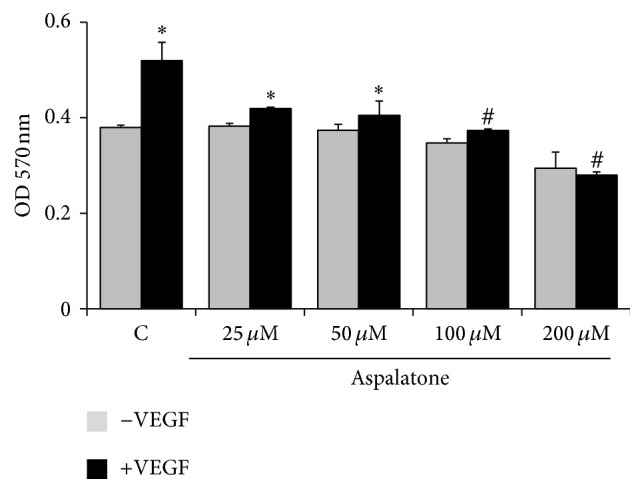
Effect of aspalatone on HAECs viability: HAECs (3000 cells/well) were treated with indicated concentrations of aspalatone without or with VEGF (10 ng/mL). After 24 h, cell viability was determined by MTT assay. Values are mean ± SD (*n* = 5). ^*∗*^*p* < 0.05 when compared with control and ^#^*p* < 0.005 when compared with VEGF- treated group.

The effect of aspalatone on VEGF-induced cell viability is not known; therefore, we first examined the effect of aspalatone on viability of HAECs. As shown in Figure 1, treatment of cells with aspalatone alone did not cause any significant increase in the cell growth or death when measured using aspalatone concentrations at 25, 50, 100, and 200 *μ*M. However, treatment of HAECs with VEGF increased cell growth and this increase was prevented by pretreatment of aspalatone. Thus, our results suggest that aspalatone alone did not show any significant effect on HAEC viability but decreased VEGF-induced increase in the cell growth.

### 3.2. Effect of Aspalatone on VEGF- Induced HAECs Migration and Tube Formation

**Figure 2:**
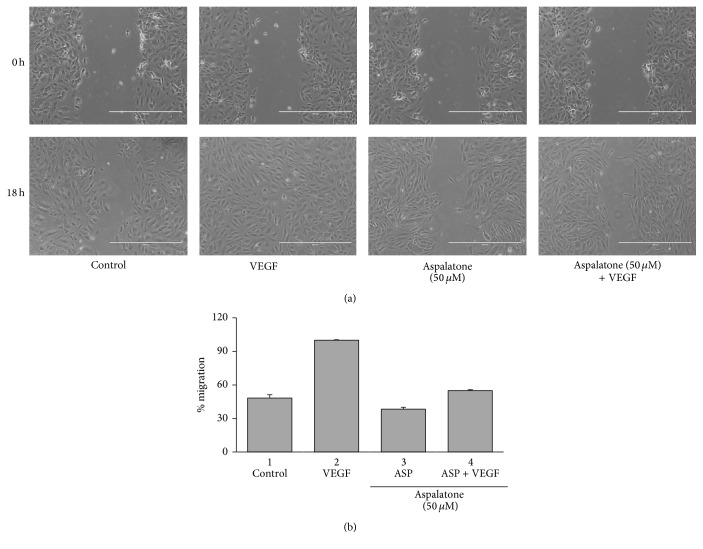
Aspalatone inhibits VEGF-induced migration of HAECs. (a) In vitro wound healing assay showing effect of aspalatone on VEGF-induced migration in HAECs. HAECs were grown in 12-well tissue culture dishes and after the cells became confluent, the monolayer was scratched with a sterile pipette tip. The media was replaced with VEGF (10 ng/mL) without or with aspalatone (50 *μ*M) and the cells were incubated for the indicated time periods. Photographs were taken at 0 h and 18 h when the VEGF containing monolayer wound was completely filled. (b) Histogram showing migration rate calculated by formula (Width_0 hr_−Width_18 hr_)/Width_0 hr_ × 100. Representative photographs from three independent experiments are shown. Magnification 10x. Scale bar: 400 *μ*m.

**Figure 3:**
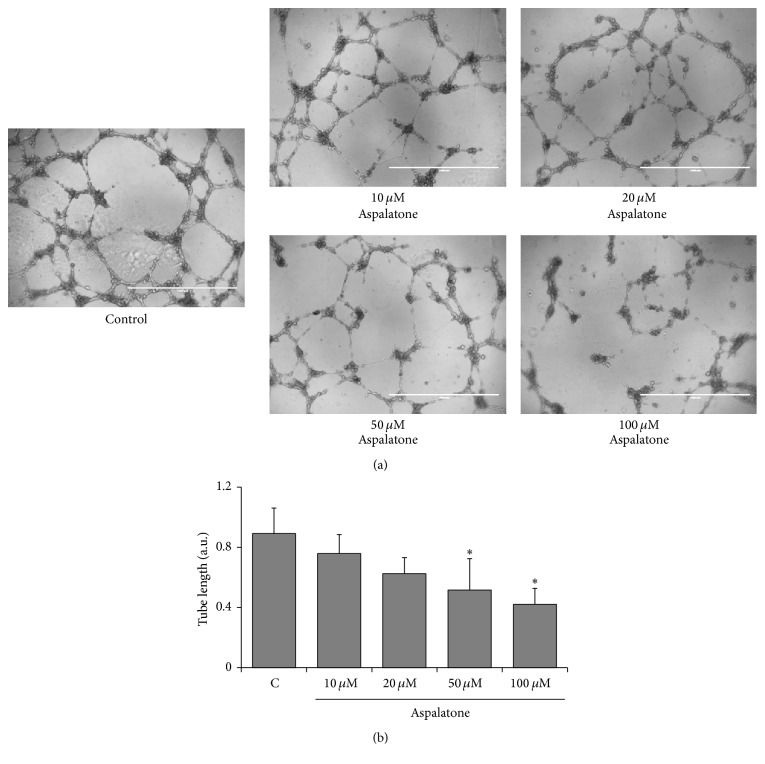
Aspalatone inhibits VEGF-induced tube formation of HAECs. (a) In vitro angiogenesis assay was performed in the absence or presence of different concentrations of aspalatone (10 *μ*M, 20 *μ*M, 50 *μ*M, and 100 *μ*M) by using in vitro angiogenesis assay kit from Millipore. (b) Histograms showing length of capillaries treated with different concentration of aspalatone. Bars represent mean ± SD (*n* = 6). Representative photographs from three independent experiments are shown. Magnification 4x. ^*∗*^*p* < 0.05 when compared with control group. Scale bar: 1000 *μ*m.

We next examined the efficacy of aspalatone in the prevention of VEGF-induced cell migration. In vitro wound healing assay was performed in cells treated with VEGF in the absence and presence of aspalatone. The results shown in Figures 2(a) and 2(b) indicate that VEGF caused migration of HAECs, which is observed by complete closure of the wound; however, pretreatment of aspalatone prevented the VEGF-induced migration of HAECs. Since migration of cells is an important step for neovascularization, we next examined the effect of aspalatone on VEGF-induced tube formation in vitro, a standard method for determining the angiogenesis in vitro. HAECs were seeded on 96-well plates coated with ECM matrix gel solution. The ECM matrix contained a mixture of growth factors, including VEGF for maximal induction of endothelial cell tube formation. As shown in Figures 3(a) and 3(b), compared to control cells, aspalatone treated cells show significant reduction in the formation of capillary like structures in a dose-dependent manner. Treatment of cells with 50 *μ*M and 100 *μ*M aspalatone significantly prevented the tube formation of endothelial cells, suggesting that aspalatone could prevent neovascularization.

### 3.3. Effect of Aspalatone on VEGF-Induced Endothelial Dysfunction

**Figure 4:**
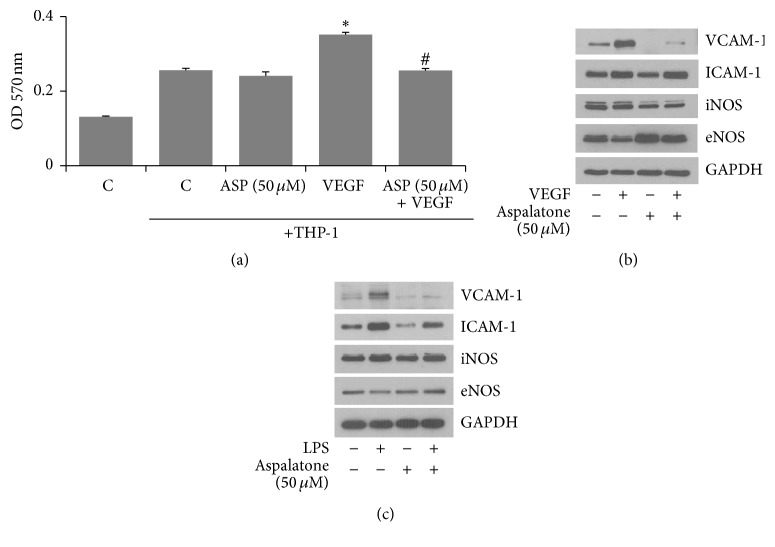
Aspalatone inhibits VEGF-induced adhesion of monocytes to HAECs. (a) HAECs (3000 cells/well) in 96-well plates were treated with aspalatone (50 *μ*M) overnight followed by addition of THP-1 cells and VEGF (10 ng/mL) for another 18 h. Cell adhesion was determined by MTT absorbance recorded at 570 nm using a plate reader. Bars represent mean ± SD (*n* = 5). ^*∗*^*p* < 0.05 when compared with control and ^#^*p* < 0.005 when compared with VEGF-treated group. HAECs treated without or with (b) VEGF (10 ng/mL) or (c) LPS (1 *μ*g/mL) in the absence and presence of aspalatone (50 *μ*M) for 18 h. Equal amounts of cell extracts were subjected to Western blot analysis using specific antibodies against VCAM-1, ICAM-1, eNOS, iNOS, and GAPDH in HAECs.

Since adhesion of monocytes to the endothelial cells is considered to be one of the initial events in vascular pathologies, we next examined the effect of aspalatone on VEGF-induced monocyte adhesion to HAECs. As shown in Figure 4(a), VEGF caused a significant increase in the adhesion of monocytes to HAECs and in the presence of aspalatone the adhesion of monocytes to HAECs significantly decreased. Since increased expression of adhesion molecules such as ICAM-1 and VCAM-1 has been shown to be responsible for monocyte adhesion, we next examined the effect of aspalatone on VEGF-induced expression of adhesion molecules in HAECs. As shown in Figure 4(b), VEGF alone induced the expression of VCAM-1 and ICAM-1 in HAECs and in cells treated with aspalatone + VEGF, and the expression of VCAM-1 but not ICAM-1 was significantly decreased. However, when compared to controls, aspalatone alone did not increase the expression of these adhesion molecules in HAECs. Thus, these results suggest that aspalatone prevents VEGF-induced monocyte adhesion by preventing the expression of adhesion molecules. We next examined the effect of aspalatone on the expression of eNOS and iNOS in endothelial cells. The data shown in Figure 4(b) suggest that VEGF caused a decrease in the expression of eNOS and increase in the expression of iNOS in the HAECs and aspalatone significantly reversed VEGF-induced changes in the expression of eNOS and iNOS suggesting that aspalatone prevents VEGF-induced endothelial dysfunction. To examine if the effect of aspalatone is not only restricted to VEGF but also to other oxidants, we next treated HAECs with lipopolysaccharides (LPS, 1 *μ*g/mL) in the presence and absence of 50 *μ*M aspalatone and examined the expression of eNOS, iNOS, VCAM-1, and ICAM-1. As shown in Figure 4(c), aspalatone also prevented LPS-induced increase in the expression of VCAM-1, ICAM-1, and iNOS in HAECs. Furthermore, aspalatone also restored the LPS-induced decrease in the eNOS levels. Thus, our results suggest that aspalatone could prevent oxidative stress-induced endothelial dysfunction.

### 3.4. Effect of Aspalatone on VEGF-Induced Expression of Inflammatory Markers

**Figure 5:**
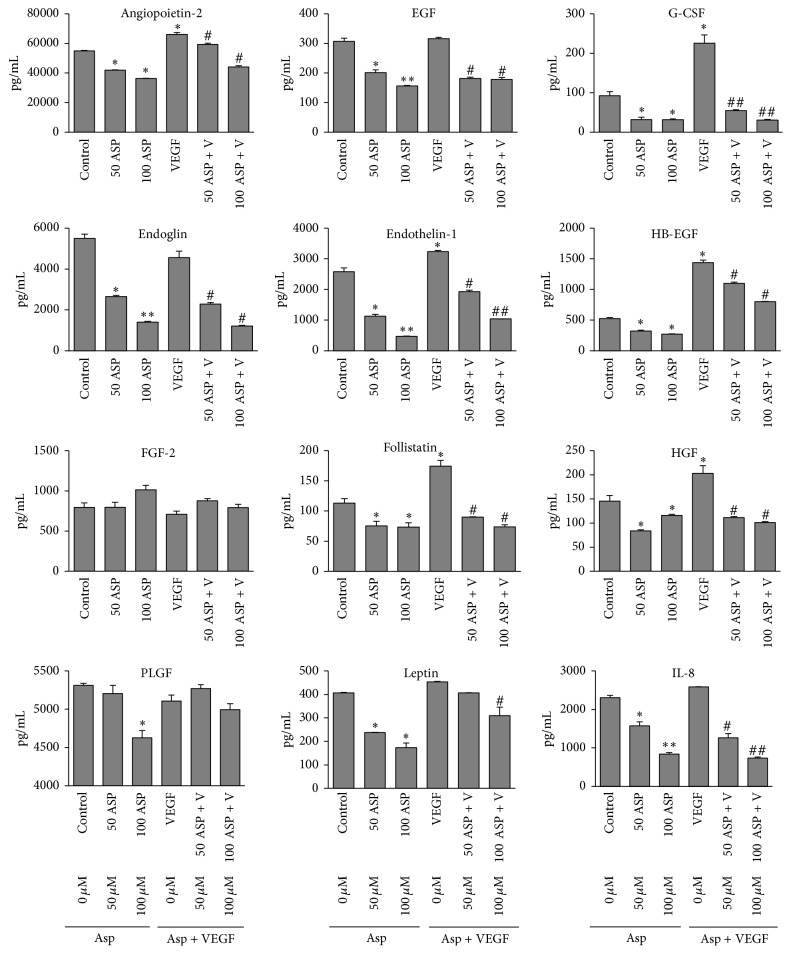
Aspalatone modulates VEGF-induced expression of inflammatory cytokines and growth factors in HAECs. Culture media of HAECs treated with VEGF (10 ng/mL) in the absence or presence of aspalatone (50 *μ*M and 100 *μ*M) were analyzed for inflammatory cytokines by using a Human Angiogenesis/Growth Factor Magnetic bead panel kit from Millipore following manufacturer's instructions using a Milliplex Analyzer System. Bars represent mean ± SD (*n* = 4). ^*∗*^*p* < 0.05 and ^*∗∗*^*p* < 0.001 when compared with control and ^#^*p* < 0.01 and ^##^*p* < 0.001 when compared with VEGF-treated group.

Since it is well known that increased expression of various cytokines and growth factors in HAECs are responsible for endothelial dysfunction, we next examined the effect of aspalatone on VEGF-induced expression of various inflammatory markers in HAECs by using a Millipore multiplex ELISA array. Results shown in Figure 5 indicate a significant increase in the expression of Angiopoietin-2, EGF, Follistatin, Leptin, G-CSF, HB-EGF, HGF, and IL-8 that were observed in the VEGF-treated HAEC culture media, and aspalatone significantly reduced the VEGF-induced release of inflammatory cytokines and growth factors in the culture media. These results suggest that aspalatone by preventing the expression of various inflammatory markers prevents VEGF-induced endothelial dysfunction and tube formation.

### 3.5. Effect of Aspalatone on VEGF-Induced ROS Formation and Lipid Peroxidation

**Figure 6:**
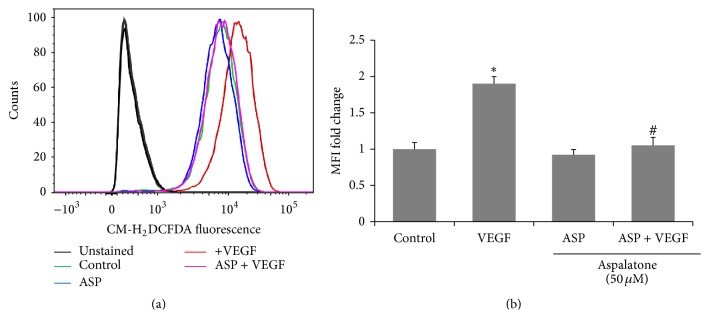
Aspalatone inhibits VEGF-induced reactive oxygen species production in HAECs. (a) Flow cytometric analysis showing CM-H_2_DCFDA fluorescence in HAECs treated with VEGF (10 ng/mL) without or with aspalatone (50 *μ*M) for 18 h (red: VEGF alone treated, pink: VEGF + aspalatone, green: control, and blue: aspalatone). Unfilled histograms with solid black lines were unstained controls. (b) Bars showing fold change in CM-H_2_DCFDA Mean Fluorescence Intensity (MFI) in HAECs treated with aspalatone (50 *μ*M) in the absence or presence of VEGF (10 ng/mL) for 18 h. Bars represent mean ± SD (*n* = 3). ^*∗*^*p* < 0.05 when compared with control and ^#^*p* < 0.001 when compared with VEGF-treated group.

**Figure 7:**
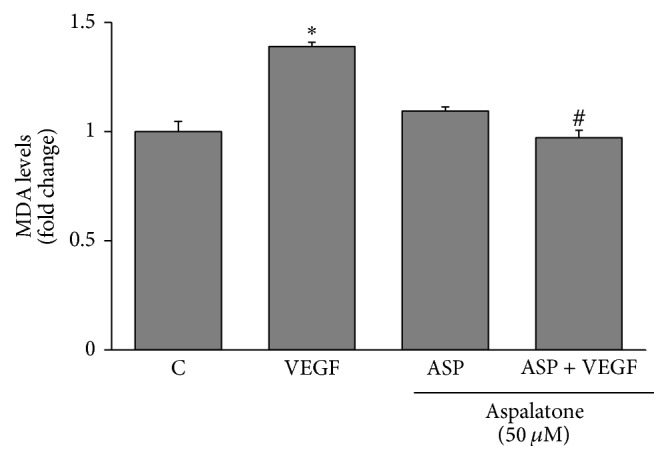
Aspalatone prevents VEGF-induced lipid peroxidation-derived malondialdehyde levels in HAECs. HAECs treated without or with 50 *μ*M aspalatone in the absence and presence of VEGF (10 ng/mL) for 18 h. The MDA levels were measured by using a kit from Oxis International Inc. MDA values (*μ*M) were normalized to total cellular protein content and presented as fold change compared to control. Bars represent mean ± SD (*n* = 3). ^*∗*^*p* < 0.05 when compared with control and ^#^*p* < 0.001 when compared with VEGF-treated group.

Oxidative stress is the major cause of VEGF-induced endothelial dysfunction and growth factors are well known to exert their effects by increasing the oxidative stress. Therefore, we next examined the effect of aspalatone on VEGF-induced generation of ROS and lipid peroxidation in HAECs. Our results shown in Figures 6(a) and 6(b) indicate an approximately 2-fold increase in the generation of ROS in cells treated with VEGF. This increase in ROS production in HAECs was significantly prevented by aspalatone. Aspalatone alone did not cause any significant changes in the ROS levels and was comparable with the untreated control cells. Since lipid peroxidation is an important event during oxidative stress and acts as an indicator of increased oxidative stress, we next examined the effect of aspalatone on VEGF-induced malondialdehyde (MDA), a marker of lipid peroxidation. The MDA levels in HAECs treated with VEGF in the absence or presence of aspalatone were determined by MDA assay kit. Our results shown in Figure 7 indicate an approximately 1.3-fold increase in the formation of MDA levels in VEGF-treated cells, and this increase was significantly prevented by the aspalatone suggesting that aspalatone prevents VEGF-induced oxidative stress in HAECs.

## 4. Discussion

Despite extensive evidence that shows the anti-inflammatory, antitumorigenic, and antioxidative activities of aspirin [48–51], to the best of our knowledge, we are not aware of any studies that have documented aspalatone in restoring the growth factor-induced endothelial dysfunction. In this study, we have provided evidence that aspalatone, an esterified derivative of aspirin and maltol, prevents VEGF-induced endothelial dysfunction. Our data suggests that the antioxidant effects of aspalatone prevented the VEGF-induced ROS, lipid peroxidation, and expression of various inflammatory markers in the endothelial cells. Thus, our data for the first time indicate that aspalatone prevents endothelial dysfunction.

Endothelial cells play an important role in atherosclerosis and other cardiovascular complications, including chronic heart diseases [1, 4, 6, 9]. Endothelial dysfunction is associated with an increased risk for CVD [6, 9]. Several studies have shown that oxidative stress-generated ROS levels dictate various cellular processes such as cell growth, differentiation, apoptosis, DNA damage, and tissue injury [52–54]. Antioxidants have attained considerable attention in this regard as various antioxidants have been reported to improve endothelial function by reducing the formation of ROS thereby preserving endothelial cell integrity and function [25–28]. Plant-derived polyphenols have also been reported to improve endothelial cell function and exert a beneficial effect on vascular pathologies [55]. Growth factors such as VEGF bind to its receptors VEGFR1 and VEGFR2 and activate downstream signaling pathways of oxidative stress mediated various kinases such as p38MAPK, AKT, and JNK, which are important regulators of endothelial cell activation [56, 57]. Chiu et al. have shown that *β*2-glycoprotein-I prevents VEGF-induced HAEC growth as well as migration by regulating the activation of ERK1/2 and AKT [58]. Further, Shu et al. have shown that vasostatin prevents endothelial cell growth by activation of caspase-3 and downregulation of eNOS in HUVEC cells [59]. Similarly, upregulation of Notch signaling by VEGF induces ROS production via activation of Rac1 dependent NADPH oxidase in endothelial cells [57, 60]. ROS in turn activate various redox-sensitive transcription factors such as HIFs, AP-1, and NF-*κβ*, which transcribe various proinflammatory genes that regulate various signaling pathways leading to endothelial cell activation and dysfunction [61, 62]. Antioxidants such as NAC, Vitamin C, and inhibitors of ROS production have been reported to be effective in preventing endothelial cell activation in culture and animal models [25–29]. In addition, aspirin has been shown to be an antioxidant with anti-inflammatory actions. Aspirin has been shown to reduce the endothelial dysfunction, hypertension, and cardiovascular complications [32, 33]. It is considered as one of the most commonly used analgesic agents that confers significant protection against inflammatory complications, including atherosclerosis, diabetes, and several forms of cancers [32–37]. Our data suggest that aspalatone inhibited VEGF-induced ROS production in HAECs indicating that VEGF-induced endothelial dysfunction may be mediated through ROS. Increased ROS could lead to subsequent reduction in the endothelial Nitric Oxide (NO) availability and decreases eNOS levels. Several studies have shown that decreased eNOS levels could lead to endothelial dysfunction [26, 63, 64]. Since eNOS is a key regulator of vascular tone and antithrombotic process, therefore, we have examined the effect of aspalatone on VEGF- and LPS-induced eNOS. Our results indicate that aspalatone restored the VEGF- and LPS-depleted eNOS levels suggesting that by restoring eNOS levels aspalatone could improve endothelial dysfunction. Consistent with our data, antioxidants such as curcumin have been shown to restore eNOS levels and improve endothelial dysfunction in vitro and in vivo [24–26].

ROS also induces lipid peroxidation and lipid peroxidation-generated aldehydes are important cellular signaling molecules that regulate various cellular processes [62, 65] including endothelial cells activation and monocyte adhesion [66–68]. Several studies have shown that oxidized lipids and lipid aldehydes such as 4-hydroxynonenal cause endothelial dysfunction leading to monocyte adhesion which play a significant role in vascular inflammation during atherosclerosis [16, 68]. Monocytes have been shown to be important regulators of immune response and play an important role in angiogenesis [69, 70]. Further, several studies have shown that neovascularization is a major contributor to atherosclerotic plaque progression [71]. Our results show that aspalatone prevented VEGF-induced lipid peroxidation-derived aldehyde, malondialdehyde in HAECs, suggesting that aspalatone prevents generation of lipid peroxidation end products responsible for vascular complications. Furthermore, our studies also indicate that aspalatone prevents VEGF-induced monocyte adhesion to endothelial cells by decreasing the VEGF-induced expression of VCAM-1 and ICAM-1. VEGF has been shown to regulate the endothelial function by regulating the expression of ICAM-1 and VCAM-1 that leads to various vascular inflammatory pathologies [72]. Radisavljevic et al. have also shown that VEGF induced endothelial cell migration by upregulating the ICAM-1 expression via PI3K/AKT/NO in brain microvascular endothelial cells [73]. Angiopoietin-1 has been shown to inhibit VEGF-induced leukocyte adhesion to endothelial cells by preventing the expression of adhesion molecules such as ICAM-1, VCAM-1, and E-selectin [74]. Various cytokines, chemokines, and growth factors like interleukins (IL-1, IL-6, and IL-8), tumor necrosis factor (TNFs), transforming growth factor beta (TGF*β*), granulocyte macrophage-colony stimulating factors (GM-CSF), and interferons have been reported to play important roles in vascular pathologies including atherosclerosis [69, 70, 75]. Various natural antioxidants have been shown to prevent the release of various proinflammatory cytokines and growth factors from endothelial cells [30, 76, 77]. Aspirin, curcumin, resveratrol, and several flavonoid compounds have been shown to control CVD because of their activity in preventing the expression of various inflammatory markers [30–33, 37, 38, 76, 77]. Our results have also demonstrated that aspalatone significantly inhibited the release of various cytokines and growth factors in endothelial cells with or without VEGF treatment. It is possible that aspalatone prevents VEGF-induced endothelial dysfunction by inhibiting the inflammatory cytokines production as well as their autocrine and paracrine crosstalk effects with VEGF.

In summary, although aspirin has also been reported to protect from CVD via its antioxidative and anti-inflammatory properties, some common side effects like gastrointestinal (GI) insult and gastric and duodenal mucosal damage have been reported [39, 40]. Conversely, aspalatone has been shown to have negligible side effects on the GI tract and exhibited similar antioxidant and antiplatelet activity as aspirin [41–45]. However, the effect of aspalatone on endothelial dysfunction is not known. Our studies showed that treatment of HAECs with aspalatone blocked VEGF-induced endothelial dysfunction by restoring the eNOS levels and inhibiting the expression of inflammatory and adhesion molecules. The mechanism of the inhibitory effect of aspalatone on VEGF-induced endothelial dysfunction may involve the antioxidant and anti-inflammatory functions of aspalatone and warrant further investigations in the therapeutic development of this compound.
